# Plant‐Derived Bioactives and PPAR Signaling in Functional Dyspepsia: Mechanistic Insights and Therapeutic Perspectives

**DOI:** 10.1002/ptr.70449

**Published:** 2026-09-13

**Authors:** Irene Palenca, Emidio Scarpellini, Jan Tack, Pierluigi De Pierro, Giovanni Sarnelli, Giuseppe Esposito

**Affiliations:** ^1^ Neuropharmacology and Behavioral Science Laboratory IRCCS Fondazione Santa Lucia Rome Italy; ^2^ Translational Research Center for Gastrointestinal Disorders (TARGID), Department of Chronic Diseases and Metabolism (CHROMETA) KU Leuven, Leuven, Belgium‐Internal Medicine Unit, Madonna del Soccorso General Hospital San Benedetto del Tronto Italy; ^3^ Translational Research Center for Gastrointestinal Disorders (TARGID), Department of Chronic Diseases and Metabolism (CHROMETA) Leuven Belgium; ^4^ Department of Clinical Medicine and Surgery University of Naples Federico II Naples Italy; ^5^ Department of Physiology and Pharmacology “V. Erspamer” Sapienza University of Rome Rome Italy

**Keywords:** duodenal barrier, functional dyspepsia, palmitoylethanolamide, phytotherapy, plant‐derived bioactives, PPAR

## Abstract

Functional dyspepsia (FD) is a common disorder of gut‐brain interaction in which gastric sensorimotor abnormalities may coexist with impaired duodenal barrier function, low‐grade immune activation, and altered neuroimmune signaling. Peroxisome proliferator‐activated receptors (PPARs) are relevant to this emerging model because they regulate epithelial differentiation, inflammatory responses, mitochondrial metabolism, redox balance, and lipid‐mediator signaling. Impaired duodenal release of palmitoylethanolamide (PEA) provides direct translational evidence that defective PPARα‐dependent lipid signaling may contribute to FD. Experimental studies further suggest that several plant‐derived bioactives modulate PPAR‐related pathways and other mechanisms involved in mucosal adaptation. Whether these effects restore duodenal function or improve clinically meaningful outcomes in humans remains uncertain. This review examines PPAR signaling as a mechanistic link between duodenal dysfunction and the actions of plant‐derived bioactives. Therefore, we distinguish disease‐specific evidence from findings obtained in other experimental settings and identify priorities for the development of mechanism‐based phytotherapy in FD.

## Graphical abstract

Functional dyspepsia involves duodenal barrier dysfunction, low‐grade immune activation and altered neuroimmune signalling. Various plant‐derived bioactives may modulate PPAR signalling, reduce inflammatory and oxidative stress, and support epithelial, immune, metabolic, and sensory homeostasis. These mechanisms could improve duodenal function and potentially relieve dyspeptic symptoms. In the graphical abstract, disease‐specific human evidence is distinguished from indirect/preclinical evidence: impaired duodenal PEA–PPARα signaling is identified as the principal patient‐supported pathway, whereas the effects of other plant‐derived bioactives on PPAR‐related epithelial, immune, metabolic, and sensory pathways are indicated as predominantly experimental or indirect.

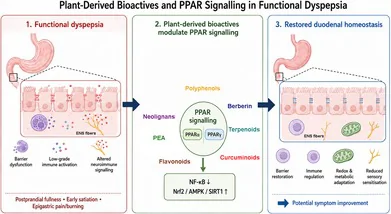

**Abbreviations**
AMPK: AMP‐activated protein kinase
AP‐1: activator protein 1
DGBI/DGBIs: disorder(s) of gut‐brain interaction
EGCG: epigallocatechin‐3‐gallate
EPS: epigastric pain syndrome
FAAH: fatty acid amide hydrolase
FD: functional dyspepsia
MAPK: mitogen‐activated protein kinase
NAAA: N‐acylethanolamine acid amidase
NAE/NAEs: N‐acylethanolamine(s)
NF‐κB: nuclear factor kappa B
Nrf2: nuclear factor erythroid 2‐related factor 2
PDS: postprandial distress syndrome
PEA: palmitoylethanolamide
PGC‐1α: peroxisome proliferator‐activated receptor gamma coactivator 1‐alpha
PPAR/PPARs: peroxisome proliferator‐activated receptor(s)
PPARα: peroxisome proliferator‐activated receptor alpha
PPARβ/δ: peroxisome proliferator‐activated receptor beta/delta
PPARγ: peroxisome proliferator‐activated receptor gamma
RCT: randomized controlled trial
ROS: reactive oxygen species
SIRT1: sirtuin 1
STW 5: standardized nine‐herb combination marketed as Iberogast
TRP: transient receptor potential
TRPA1: transient receptor potential ankyrin 1
TRPV1: transient receptor potential vanilloid 1
TRPV4: transient receptor potential vanilloid 4
ZO‐1: zonula occludens 1

## 1 Introduction

Functional dyspepsia (FD) is among the most prevalent disorders of gut‐brain interaction (DGBIs), affecting approximately 5%–10% of adults worldwide and accounting for a substantial proportion of gastroenterology referrals (Talley and Ford 2015; Enck et al. 2017). Despite this burden, treatment remains largely empirical and benefits only a subset of patients. Rome IV classified FD as a DGBI and defined postprandial distress syndrome (PDS) and epigastric pain syndrome (EPS), while acknowledging their frequent overlap (Stanghellini et al. 2016). Rome V places greater emphasis on the interplay among gastrointestinal physiology, epithelial integrity, mucosal immunity, microbial ecology, and gut‐brain communication, supporting a shift towards mechanism‐based, biomarker‐guided treatment (Drossman et al. 2026; Camilleri et al. 2026).

This shift is particularly relevant to FD because similar symptoms may arise from different biological mechanisms. Delayed gastric emptying, impaired accommodation, and visceral hypersensitivity remain established features, but they account for only part of the clinical phenotype and correlate inconsistently with treatment response (Tack et al. 1998; Camilleri 2006). Attention has therefore broadened from gastric sensorimotor function to the duodenal mucosa. The duodenum is increasingly recognized as an important site of disease expression, where barrier dysfunction, low‐grade immune activation, and altered neuroimmune signaling may converge (Vanheel et al. 2014; Wauters et al. 2021; Du et al. 2018). These abnormalities support a model of disrupted duodenal homeostasis rather than a collection of unrelated lesions (Huyghe et al. 2025). This peripheral perspective does not diminish the contribution of central pain processing, autonomic dysfunction, or psychosocial factors, all of which remain integral to FD pathophysiology (Tack et al. 2006; Drossman et al. 2026).

This review focuses on the gastroduodenal compartment because it provides a plausible link among epithelial stress, endogenous lipid mediators, and PPAR signaling. PPARs regulate several processes relevant to mucosal adaptation, including immune control, oxidative responses, mitochondrial metabolism, and lipid signaling (Michalik et al. 2006; Kersten 2014). Direct translational support in FD comes from evidence of impaired duodenal PEA signaling (Sarnelli et al. 2021). Several plant‐derived bioactives, including curcuminoids, flavonoids, stilbenes, alkaloids, and terpenoids, act on PPAR‐related and complementary pathways (Villarroel‐Vicente et al. 2021; Enayati et al. 2022). Throughout this review, “plant‐derived bioactives” is used as an inclusive term for classical phytochemicals and plant‐associated/dietary bioactive molecules discussed in relation to PPAR signaling. PEA is considered separately within this framework: it is primarily an endogenous N‐acylethanolamine, but its occurrence in botanical and dietary matrices makes it a useful bridge between endogenous lipid‐mediator biology and nutraceutical/phytotherapeutic concepts. However, clinical studies have generally focused on symptom relief without establishing whether these compounds restore biological pathways implicated in FD. Their mechanisms of benefit therefore remain uncertain. Accordingly, we consider PPAR signaling as a testable framework rather than an established primary defect in FD. Compounds are discussed according to the biological functions they influence and the strength of evidence supporting their relevance to the human gastroduodenal mucosa.

Literature search and selection. As this is a narrative review, the literature was identified through targeted searches of PubMed/MEDLINE, Scopus, and Web of Science, covering publications available up to July 2026. Search terms included combinations of “functional dyspepsia,” “duodenum,” “PPAR,” “PPARalpha,” “PPARgamma,” “PPARbeta/delta,” “plant‐derived bioactives,” “phytochemicals,” “curcumin,” “quercetin,” “berberine,” “resveratrol,” “ginger,” “peppermint,” “caraway,” and “palmitoylethanolamide.” Priority was given to human FD studies, studies using human gastroduodenal tissue, randomized or controlled clinical studies, and mechanistic experimental work directly relevant to epithelial, immune, neuroimmune, metabolic, redox, or lipid‐mediator homeostasis. Additional references were identified from relevant reviews and reference lists. The search was intended to support a mechanistic narrative synthesis rather than a systematic review or meta‐analysis.

## 2 From Pathophysiological Mechanisms to Biological Integration in Functional Dyspepsia

The pathophysiological model of FD has evolved from one centred on isolated gastric dysfunction to a broader view of a heterogeneous DGBI. Gastric sensorimotor abnormalities remain relevant, but they coexist with impaired mucosal integrity, immune activation, and altered gut‐brain signaling (Talley and Ford 2015; Drossman et al. 2026). The duodenum is now recognized as an active interface integrating acid, bile acids, nutrients, food antigens, and microbial metabolites. Impaired barrier function, abnormalities of epithelial junctions, and low‐grade inflammation have been reported in FD (Vanheel et al. 2014), together with increased numbers of duodenal eosinophils and mast cells in selected cohorts (Shah et al. 2022). The observation that proton‐pump inhibition can improve symptoms while reducing permeability and mucosal immune‐cell infiltration further supports the clinical relevance of these findings (Wauters et al. 2021). Together, they position the duodenal mucosa as a key interface between luminal exposure and sensory signaling. The crucial question is how these abnormalities interact. PPARs are plausible regulators because they influence epithelial adaptation, inflammatory transcription, cellular metabolism, redox responses, and lipid‐mediator signaling (Michalik et al. 2006). Current evidence does not establish a primary or generalized defect in PPAR function in FD; rather, it supports a mechanistic hypothesis that requires disease‐specific validation.

## 3 PPAR Signalling as a Regulator of Gastrointestinal Homeostasis

Gastrointestinal homeostasis depends on the continuous adaptation of epithelial, immune, metabolic, and neuronal systems to acid, nutrients, bile acids, microbial metabolites, and mechanical stimuli. When these responses are inadequate, mucosal resilience may decline even in the absence of overt inflammation. PPARs are ligand‐activated nuclear receptors that contribute to this adaptive response.

Initially characterised as regulators of lipid metabolism, PPARs are now recognised as sensors that integrate nutritional, metabolic, inflammatory, and environmental signals (Michalik et al. 2006; Kersten 2014). Three isoforms have been identified: PPARα, PPARβ/δ, and PPARγ (Kota et al. 2005). Their functions overlap but are not identical. PPARα is closely linked to fatty‐acid oxidation, metabolic adaptation, and endogenous lipid mediators. PPARγ is expressed in intestinal epithelial and immune cells, where it supports epithelial differentiation, microbial tolerance, and the control of inflammatory responses (Dubuquoy et al. 2003). PPARβ/δ has recognised roles in lipid metabolism, cellular differentiation, and inflammatory regulation in other tissues and gastrointestinal settings, but the evidence base in the upper gastrointestinal tract is substantially smaller than that for PPARα and PPARγ. Importantly, we are not aware of disease‐specific studies demonstrating altered PPARβ/δ signaling in functional dyspepsia. For these reasons, PPARβ/δ will not be discussed in detail here, except where relevant to individual bioactives or pathway‐level observations. Through transactivation and transrepression, PPARs influence barrier integrity, mitochondrial metabolism, redox balance, innate immunity, and lipid‐mediator signalling. PPARγ can restrain macrophage activation and inflammatory transcription involving NF‐κB and AP‐1 (Ricote et al. 1998; Glass and Saijo 2010), whereas both PPARα and PPARγ interact with AMPK, Nrf2, SIRT1, and endogenous lipid mediators (Enayati et al. 2022). These functions overlap with processes implicated in FD, but this convergence remains indirect and should not be interpreted as evidence that FD is caused by PPAR dysfunction (Figure 1).

**FIGURE 1:**
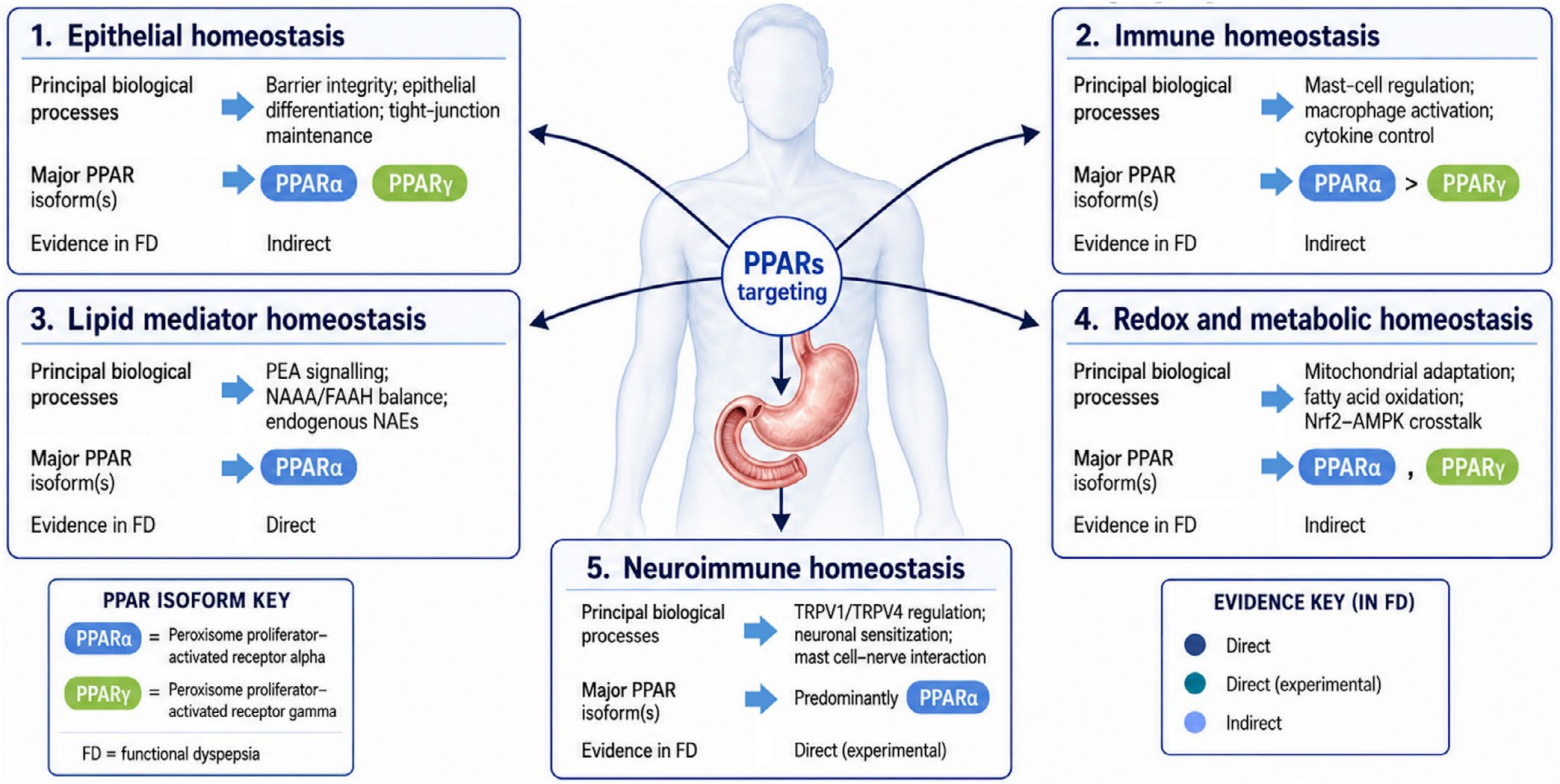
Major homeostatic functions regulated by PPAR signaling and their potential relevance to functional dyspepsia.

The strongest direct link between PPAR signaling and FD involves PEA, an endogenous N‐acylethanolamine produced in response to tissue stress. PEA limits mast‐cell activation and nociceptive signaling, partly through PPARα (Lo Verme et al. 2005; Skaper et al. 2015). In human duodenal biopsies, acid exposure induced less PEA release in patients with FD than in healthy controls. This defect was accompanied by greater mast‐cell activation and enhanced TRPV1/TRPV4‐associated neuronal sensitization, whereas restoration of PEA signaling attenuated both responses through a PPARα‐dependent mechanism (Sarnelli et al. 2021). These findings converge toward failure of an endogenous protective response rather than simply an increase in downstream inflammation. Although they do not establish a general defect in PPAR signaling in FD, they provide a disease‐specific rationale for investigating whether PPAR‐related pathways can be therapeutically reinforced. Table 1 summarizes the relevant biological domains and the strength of evidence in FD.

**TABLE 1:** Major homeostatic functions regulated by PPAR signalling and their relevance to functional dyspepsia.

Homeostatic domain	Principal biological processes	Major PPAR isoform(s)	Evidence in FD	References
Epithelial homeostasis	Barrier integrity, epithelial differentiation, tight‐junction maintenance	PPARα, PPARγ	Indirect	Dubuquoy et al. 2003; Vanheel et al. 2014
Immune homeostasis	Mast‐cell regulation, macrophage activation, cytokine control	PPARγ > PPARα	Indirect	Ricote et al. 1998; Wauters et al. 2021
Lipid mediator homeostasis	PEA signalling, NAAA/FAAH balance, endogenous NAEs	PPARα	Direct	Sarnelli et al. 2021
Redox and metabolic homeostasis	Mitochondrial adaptation, fatty‐acid oxidation, Nrf2–AMPK crosstalk	PPARα, PPARγ	Indirect	Michalik et al. 2006; Kersten 2014
Neuroimmune homeostasis	TRPV1/TRPV4 regulation, neuronal sensitization, mast cell–nerve interaction	Predominantly PPARα	Direct (experimental)	Sarnelli et al. 2021

## 4 Plant‐Derived Bioactives as Modulators of PPAR‐Dependent Homeostatic Regulation

### 4.1 From Homeostatic Regulation to Network Pharmacology

Current pharmacological treatment of FD targets acid secretion, motility, or pain processing, with modest and variable benefit (Ford et al. 2021; Camilleri et al. 2026). Plant‐derived bioactives are of interest because many influence PPAR signalling alongside inflammatory, antioxidant, and metabolic pathways (Huang et al. 2005; Enayati et al. 2022). Their evaluation should therefore extend beyond symptom relief to include target engagement and disease‐relevant biological outcomes. Plant‐derived bioactives commonly affect several molecular targets, including PPARs, NF‐κB, Nrf2, AMPK, and SIRT1. The activity of a phytocomplex may also reflect additive or synergistic interactions among its constituents (Williamson 2001; Panossian et al. 2022). Although this pleiotropy complicates attribution of effect, it may be particularly relevant to a multifactorial disorder such as FD. Experimental studies should establish whether PPAR signalling is required for a given effect and whether the intervention improves epithelial, immune, sensory, or metabolic function under conditions relevant to the gastroduodenal mucosa. The following sections describe the evidence by biological domain rather than botanical class. Figure 2 summarizes the reported interactions between selected plant‐derived bioactives and PPAR isoforms, while Table 2 provides an overview of their PPAR‐related mechanisms and potential relevance to FD.

**FIGURE 2:**
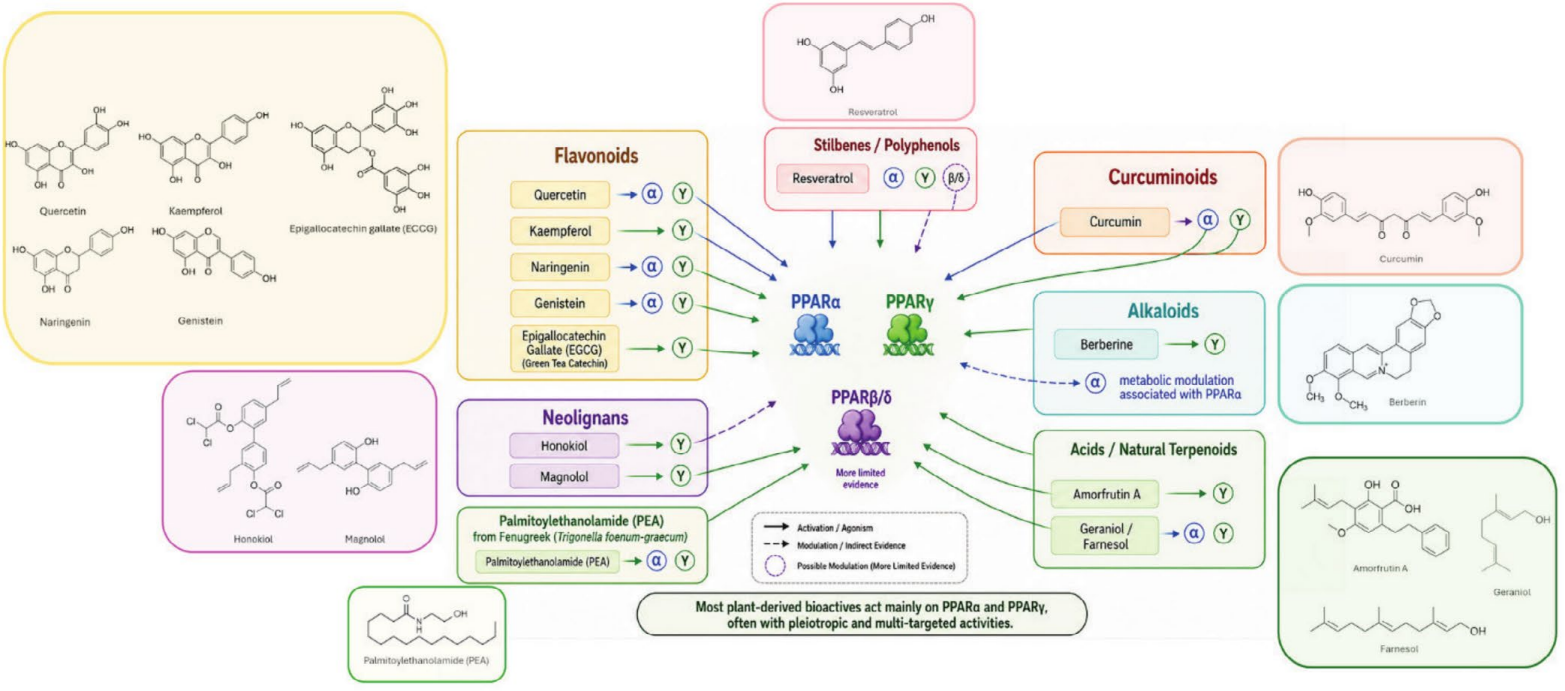
Reported interactions between selected plant‐derived bioactives and PPAR isoforms. Solid arrows indicate evidence of direct receptor activation; dashed arrows indicate indirect or pathway‐level modulation; dotted outlines denote preliminary or context‐dependent evidence. Most compounds converge on PPARα‐ and/or PPARγ‐related pathways, whereas evidence for PPARβ/δ is less consistent.

**TABLE 2:** Plant‐derived bioactives with potential to modulate PPAR‐related homeostatic pathways relevant to functional dyspepsia.

Class	Representative compounds	PPAR target(s)	Type of interaction	Potential therapeutic relevance to FD	Potential site of action	References
Flavonoids and catechins	Quercetin, kaempferol, naringenin, genistein, EGCG	PPARα and PPARγ; context‐dependent nuclear‐receptor modulation	Direct or indirect modulation, depending on compound and context	May reduce oxidative and inflammatory stress, preserve tight‐junction integrity and support epithelial repair.	Gastric and duodenal epithelium; mucosal immune cells; enteric and extrinsic sensory pathways	Avior et al. 2013; Boots et al. 2008; Suzuki and Hara 2009; Yang et al. 2025
Stilbenes and polyphenols	Resveratrol	Mainly PPARα and PPARγ, with AMPK–SIRT1–PGC‐1α crosstalk	Predominantly indirect network modulation; possible receptor‐dependent effects	May improve mitochondrial function, antioxidant defence and mucosal adaptation to postprandial stress.	Gastroduodenal epithelium; mucosal immune cells; enteric nervous system	Lagouge et al. 2006; Enayati et al. 2022
Curcuminoids	Curcumin	PPARγ and PPARα‐related pathways	Pleiotropic modulation rather than selective agonism	May limit NF‐κB‐driven inflammation, oxidative injury and epithelial‐barrier dysfunction.	Gastric and duodenal mucosa; epithelial cells; lamina propria immune cells	Aggarwal and Harikumar 2009; Hewlings and Kalman 2017; Kongkam et al. 2024
Alkaloids	Berberine	Mainly PPARγ‐related signalling; indirect PPARα and AMPK modulation	Direct and indirect network modulation	May support barrier integrity, metabolic regulation and inflammatory control, with context‐dependent effects on motility.	Intestinal epithelium; enteric neurons; smooth muscle; gut microbiota	Cao et al. 2022; Yang et al. 2023; Enayati et al. 2022
Neolignans	Honokiol, magnolol	Mainly PPARγ; possible PPARβ/δ involvement	Reported receptor activation and broader indirect modulation	May reduce mucosal inflammation, oxidative stress and neuronal hyperexcitability.	Gastric and duodenal mucosa; mucosal immune cells; enteric and visceral sensory neurons	Villarroel‐Vicente et al. 2021; Enayati et al. 2022
Plant‐associated bioactive lipids	Palmitoylethanolamide (PEA)	Predominantly PPARα	Endogenous agonism and homeostatic modulation	May restrain mast‐cell activation, neuroimmune sensitization and TRPV‐mediated nociception.	Duodenal mucosa; mast cells; enteric neurons, glia and visceral afferents	Lo Verme et al. 2005; Skaper et al. 2015; Sarnelli et al. 2021
Natural acids and terpenoids	Amorfrutin A, geraniol, farnesol	Mainly PPARγ; possible combined PPARα/γ modulation	Direct activation for selected compounds; otherwise, indirect evidence	May promote anti‐inflammatory, metabolic and antioxidant responses, although evidence remains preclinical.	Gastroduodenal epithelium; mucosal immune cells; enteric nervous system	Huang et al. 2005; Villarroel‐Vicente et al. 2021; Enayati et al. 2022

### 4.2 Restoration of Epithelial Homeostasis

The duodenal epithelium serves both as a physical barrier and as a signaling interface for nutrient sensing, immune surveillance, and neuronal communication. Increased permeability, altered junctional proteins, and low‐grade inflammation are amongst the most reproducible mucosal findings in FD (Vanheel et al. 2014; Wauters et al. 2021). Restoring barrier function may therefore represent a therapeutic objective rather than merely a consequence of symptom improvement. Epithelial resilience depends on tight‐junction dynamics, membrane composition, mitochondrial metabolism, redox control, and innate immune signaling. Curcumin influences NF‐κB, Nrf2, and PPAR‐related pathways and preserves tight‐junction proteins in experimental models (Aggarwal and Harikumar 2009; Hewlings and Kalman 2017). In a randomized, double‐blind trial in FD, curcumin improved symptoms to a degree comparable with omeprazole, but barrier, immune, and molecular biomarkers were not assessed (Kongkam et al. 2024). The mechanism underlying this clinical benefit therefore remains unclear. Berberine can activate AMPK‐related pathways, support tight‐junction expression, and reduce permeability in preclinical models, although direct evidence in the human gastroduodenal mucosa is lacking (Cao et al. 2022). EGCG suppresses NF‐κB signaling, promotes Nrf2‐associated responses, and preserves epithelial integrity in experimental systems (Gao et al. 2023; Yang et al. 2025; Rosenthal et al. 2025). Quercetin and related flavonoids also affect junctional proteins and nuclear‐receptor signaling (Boots et al. 2008; Suzuki and Hara 2009; Avior et al. 2013). In addition, quercetin produces concentration‐dependent relaxation of isolated human gastric smooth muscle through ATP‐sensitive potassium channels, providing human tissue‐based support for a potential role in dyspeptic motor dysfunction, although this observation was not obtained in patients with FD (Modzelewska et al. 2021). These findings justify further investigation of epithelial effects, but they do not yet demonstrate PPAR‐mediated restoration of barrier function in FD.

### 4.3 Restoration of Neuroimmune Homeostasis

Neuroimmune signaling is central to current models of FD. Epithelial cells, resident immune cells, enteric neurons, and extrinsic afferents form a functional unit that interprets luminal stimuli. Disruption of this unit may generate symptoms even in the absence of overt inflammation (Talley and Ford 2015; Wauters et al. 2021; Camilleri et al. 2026).

Mast cells and eosinophils contribute not only to inflammation but also to epithelial defence, tissue remodeling, and neuronal signaling. Their proximity to mucosal nerves allows bidirectional communication through histamine, proteases, cytokines, lipid mediators, and neurotrophic factors. Persistent activation may lower sensory thresholds and amplify responses to physiological stimuli (Barbara et al. 2004; Ceulemans et al. 2022).

PPARα contributes to the regulation of lipid mediators and nociception, whereas PPARγ modulates immune‐cell activation and epithelial‐immune communication (Ricote et al. 1998; Dubuquoy et al. 2003; Lo Verme et al. 2005). The disease‐specific evidence for impaired PEA‐PPARα signaling is discussed in Section 3 and is not repeated here. Among plant‐derived compounds, curcumin can reduce inflammatory signaling and mast‐cell activation in experimental systems (Aggarwal and Harikumar 2009). Luteolin inhibits mast‐cell activation and mediator release through calcium‐dependent, NF‐κB, and MAPK pathways (Kempuraj et al. 2008; Che et al. 2020; Hao et al. 2022); its interaction with PPARγ is context‐dependent and should not be interpreted as selective agonism (Puhl et al. 2012). Ginger phenolics, including 6‐gingerol and 6‐shogaol, modulate TRPV1 and TRPA1 and attenuate inflammatory and barrier‐disrupting signals in experimental models (Iwasaki et al. 2006; Kim et al. 2016; Luettig et al. 2016; Yin et al. 2019). Clinical trials in FD have rarely included neuroimmune endpoints. Future studies should combine symptom assessment with measures of duodenal permeability, immune‐cell activation, N‐acylethanolamine metabolism, and TRP‐channel expression or activity.

### 4.4 Restoration of Redox and Metabolic Homeostasis

Although their contribution to FD remains poorly defined, redox and metabolic adaptation support mucosal integrity. Persistent oxidative imbalance can disrupt tight junctions, impair cellular energy metabolism, and amplify inflammatory signaling (Jones and Neish 2017). The relevant defect may therefore be not simply an excess of reactive oxygen species, but a failure of the mechanisms that maintain redox and metabolic control during repeated luminal stress.

PPARα and PPARγ interact with AMPK, SIRT1, PGC‐1α, and Nrf2, linking substrate utilization, mitochondrial function, antioxidant defense, and inflammatory transcription (Michalik et al. 2006; Kersten 2014). Resveratrol activates SIRT1‐ and PGC‐1α‐associated programs and promotes mitochondrial biogenesis in experimental models (Lagouge et al. 2006; Valero 2014). Berberine and several flavonoids also influence AMPK‐, Nrf2‐, and PPAR‐related pathways (Cao et al. 2022; Avior et al. 2013; Enayati et al. 2022). These findings support a potential role in metabolic adaptation rather than simple free‐radical scavenging. However, evidence in FD is almost entirely indirect, and human studies should include mucosal redox and mitochondrial biomarkers before definitive therapeutic conclusions are drawn.

### 4.5 Restoring Lipid‐Mediator Homeostasis: PEA As a Bridge From Endogenous Signalling to Therapeutic Innovation

Lipid mediators regulate epithelial responses, immune activity, neuronal excitability, and tissue repair (Izzo and Sharkey 2010; Di Marzo and Silvestri 2019). The endocannabinoidome comprises endocannabinoids and related mediators, their biosynthetic and degradative enzymes, and targets such as cannabinoid receptors, TRP channels, and PPARs (Di Marzo and Silvestri 2019; Veilleux et al. 2019). PEA is an N‐acylethanolamine synthesized locally in mammalian tissues during stress and is also present in several botanical and dietary matrices, including soy‐derived products, legumes, cereals, nuts, and coffee (Petrosino 2020; Clayton et al. 2021). In plants, PEA and related N‐acylethanolamines participate in stress and protection signaling (Tripathy et al. 1999; van der Stelt et al. 2000). Its disease‐specific relevance to FD is detailed in Section 3. Here, PEA is important chiefly as a conceptual bridge between endogenous lipid regulation and phytotherapy. Its presence in foods does not establish that dietary intake reproduces the tissue concentrations, pharmacokinetics, or biological effects of a therapeutic formulation. Similarly, current evidence does not show that other plant‐derived bioactives restore duodenal PEA synthesis or inhibit its degradation in FD. This distinction should guide future pharmacological and clinical studies.

### 4.6 Translational Perspective: Plant‐Derived Bioactives as Network Modulators

The available evidence does not support a simple one‐compound‐one‐target model for plant‐derived bioactives. Many compounds influence PPARs alongside inflammatory, antioxidant, metabolic, or sensory pathways, and these effects vary according to dose, formulation, tissue, and experimental context (Hopkins 2008). For meaningful translation, receptor‐binding or reporter assays should be complemented by experiments that test causal dependence on PPAR signaling and assess disease‐relevant functions in the gastroduodenal mucosa. Standardization of extracts, bioavailability, and pharmacokinetic exposure are equally important, because mechanistic plausibility alone does not ensure clinically relevant target engagement.

Clinical evidence remains limited. Most studies in FD have assessed symptoms without measuring epithelial integrity, mucosal immune activation, endogenous lipid mediators, or PPAR‐pathway activity. Biomarker‐enriched trials are therefore needed to determine whether clinical improvement is accompanied by a reproducible biological effect and to identify subgroups most likely to respond. Table 3 summarizes the limited clinical evidence. Curcumin, STW 5, and peppermint‐caraway preparations have shown symptomatic benefit in selected studies, whereas findings for ginger are less consistent. Among classical plant‐derived bioactives, curcumin and standardized peppermint–caraway preparations currently have the clearest clinical foothold for mechanism‐enriched proof‐of‐concept studies, while quercetin is an attractive mechanistic candidate because of its direct effects on human gastric smooth muscle. PEA remains a particularly informative comparator and translational bridge because the PEA–PPARα axis is supported by disease‐specific human FD data, although PEA should not be considered a conventional phytochemical. None of these interventions has yet been shown to improve FD through a defined PPAR‐dependent mechanism. This remains the main gap between experimental pharmacology and clinical phytotherapy.

**TABLE 3:** Clinical studies of plant‐derived bioactives in functional dyspepsia.

Intervention	Clinical evidence	Mechanistic evidence	Homeostatic domain potentially affected	Main limitation
Curcumin	Positive 3‐arm RCT; *n* = 206 randomized (151 completed); 28‐day treatment; curcumin 2 × 250 mg capsules four times daily, alone or with omeprazole (Kongkam et al. 2024)	None	Epithelial/neuroimmune	No biomarkers
STW5	Positive placebo‐controlled RCT; *n* = 315; 8 weeks; liquid STW 5, 20 drops three times daily (von Arnim et al. 2007)	None	Multiple	Mechanism unknown
Ginger	Mixed/limited; combination RCT *n* = 126; 4 weeks; ginger–artichoke extract, two capsules/day (Giacosa et al. 2015); contribution of ginger alone cannot be isolated	Limited	Neuroimmune	No mechanistic endpoints
Peppermint–caraway	Positive placebo‐controlled RCT; *n* = 96; 28 days; enteric‐coated 90 mg peppermint oil +50 mg caraway oil, one capsule twice daily (May et al. 2000)	None	Sensorimotor	Mechanism unknown

## 5 Towards Mechanism‐Based Precision Phytotherapy in Functional Dyspepsia

Management of FD remains largely symptom‐driven, and treatment response varies considerably among patients. A precision‐phytotherapy strategy should combine validated clinical outcomes with biomarkers selected according to the proposed mechanism, including epithelial permeability, mast‐cell and eosinophil activation, N‐acylethanolamine metabolism, TRP‐channel sensitization, redox adaptation, and PPAR‐pathway activity. Patient‐derived organoids, organ‐on‐chip systems, spatial profiling, metabolomics, and lipidomics may help define biologically meaningful endotypes. Because gastric sensorimotor dysfunction, visceral hypersensitivity, mucosal impairment, immune activation, and central dysregulation contribute differently across patients, no single intervention is likely to be universally effective (Talley and Ford 2015; Enck et al. 2017; Ford et al. 2020). The priority is therefore to match standardized compounds or phytocomplexes to defined abnormalities and demonstrate concordant clinical and biological responses.

## 6 Conclusions

Functional dyspepsia reflects variable contributions from gastric sensorimotor dysfunction, mucosal impairment, immune activation, and altered gut‐brain signaling. PPARs regulate several processes relevant to this biology, but disease‐specific evidence remains limited. PEA provides the clearest proof of concept that failure of an endogenous PPARα‐dependent pathway may contribute to FD. For other plant‐derived bioactives, the proposed mechanisms are supported mainly by preclinical or indirect evidence. Progress towards precision phytotherapy will require standardized interventions and biomarker‐enriched trials capable of demonstrating both clinical benefit and mechanism‐consistent changes in the gastroduodenal mucosa.

## Author Contributions

**Irene Palenca:** conceptualization, writing – review and editing. **Emidio Scarpellini:** conceptualization, writing – original draft, supervision. **Jan Tack:** supervision. **Pierluigi De Pierro:** visualization, validation. **Giovanni Sarnelli:** supervision, writing – original draft, conceptualization, writing – review and editing. **Giuseppe Esposito:** writing – original draft, supervision, conceptualization, writing – review and editing.

## Funding

The authors have nothing to report.

## Ethics Statement

The authors have nothing to report.

## Consent

The authors have nothing to report.

## Conflicts of Interest

The authors declare no conflicts of interest.

## Data Availability

Data sharing not applicable to this article as no datasets were generated or analysed during the current study.
